# Canadian Covid-19 pandemic public health mitigation measures at the province level

**DOI:** 10.1038/s41597-023-02759-y

**Published:** 2023-12-08

**Authors:** Anna Akanteva, David W. Dick, Shirin Amiraslani, Jane M. Heffernan

**Affiliations:** Modelling Infection & Immunity Lab, Centre for Disease Modelling, Mathematics & Statistics, York University, 4700 Keele Street, Toronto, M3J 1P3 Ontario Canada

**Keywords:** Infectious diseases, Applied mathematics

## Abstract

The Covid-19 pandemic has prompted governments across the world to enforce a range of public health interventions. We introduce the Covid-19 Policy Response Canadian tracker (CPRCT) database that tracks and records implemented public health measures in every province and territory in Canada. The implementations are recorded on a four-level ordinal scale (0–3) for three domains, (Schools, Work, and Other), capturing differences in degree of response. The data-set allows the exploration of the effects of public health mitigation on the spread of Covid-19, as well as provides a near-real-time record in an accessible format that is useful for a diverse range of modeling and research questions.

## Background & Summary

Since January 30, 2020, SARS-CoV-2 has been declared by WHO (World Health Organization) as a virus of international concern that causes Covid-19 disease^1^. To help curb community transmission, manage the spread of the SARS-CoV-2 virus, and minimize healthcare demand and death, governments across the world have implemented a wide range of public health restrictions. In Canada, federal, provincial, and municipal governments, with the advice of Canadian health experts including the Public Health Agency of Canada (PHAC), have provided guidance on the implementation of non-pharmaceutical interventions (NPIs), with application to congregate living settings, child settings, businesses, events, and gatherings. The NPIs implemented include, but are not limited to, school closures; restrictions on gatherings and travelling; and closures of non-essential businesses. The implementation of restrictions has varied across Canadian provinces due to differences in the intensity of Covid-19 infection and other jurisdiction-specific considerations^2,3^.

We introduce Covid-19 Policy Response Canadian tracker (CPRCT) that collects publicly available information on NPIs in every province and territory in Canada, starting from January 2020. The data described in this manuscript is available from Harvard Dataverse^4^ and the actively updated tracker is available through the GitHub repository (https://github.com/ddick8/Covid-19-Policy-Response-Canadian-tracker/github.com/ddick8/Covid-19-Policy-Response-Canadian-tracker). The NPIs are recorded on a four-level ordinal scale (0–3) for three policy indicators: schools, work, and other.

The goal of this dataset is to provide a daily record of the public health measures implemented to facilitated mathematical modeling of the COVID-19 pandemic in Canada. In response to the pandemic, it is acknowledged that public health policies were not always uniformly applied throughout entire provinces. When discrepancies in the application of public health policies across provinces have arisen, we have made the decision to assign greater weight to population centers. This decision is based on the objective of providing increased representation to the areas where the number of COVID-19 cases and new infections are most prevalent. By emphasizing population centers in our analysis, we aim to allow the dataset reflect the impact and dynamics of the pandemic in the regions where the burden of the virus is highest.

CPRCT captures differences in degree of response during the Covid-19 pandemic. Manual collection and continuous validation of data ensure the high quality of CPRCT database. Our database may serve as a valuable resource for the Canadian modelling and research community as well as other researchers elucidating the impacts of public health mitigation on the Covid-19 pandemic^5,6^. Categorization of public health interventions into three groups can be beneficial for mathematical modelling activities and projects because it eliminates the problem of redundancy and overfitting. Having three variables also makes the models easier to understand and optimize.

The three categories for work, school, and other locations enable mathematical modelers to align the implemented public health interventions with age-structured contact estimates from^7^. This careful consideration of the estimated contact structure in Canada works effectively in conjunction with mathematical models, facilitating the integration of realistic contact patterns into the modeling process.

Additionally, we provide a comprehensive list of NPIs implemented for every province and territory in Canada in a separate excel document. This allows CPRCT users to develop their own categorizations for each public health measure at work, school, and other locations. The spreadsheet can also be used to validate the 0–3 categorization provided by the CPRCT team.

The CPRCT can be used to understand the effects of public health policies on the burden of Covid-19 across Canada and to quantify the effectiveness of the public health mitigation measures. It can also assist policymakers and Canadian health experts in understanding the strategies that were implemented over the pandemic, and thus the CPRCT can be used to inform future policies and restrictions against Covid-19, and other emerging infectious diseases.

**Fig. 1:**
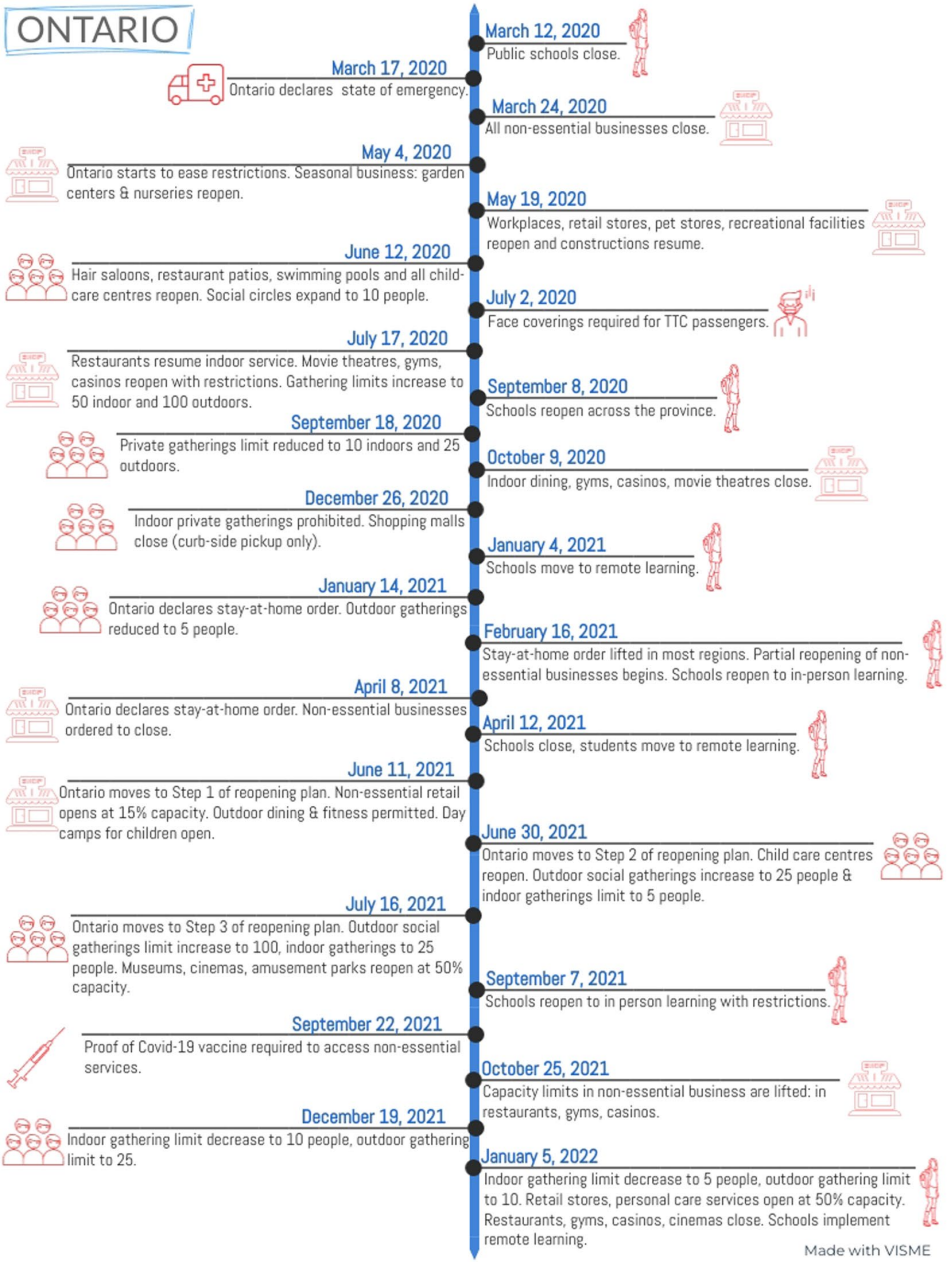
Timeline for the province of Ontario. The figure summarizes NPIs implemented from the start of the pandemic, March 12, 2020, to January 5, 2022. The timeline includes illustrations that help to discriminate between different categories of restrictions that either represent school, work, gatherings, mask mandates, and vaccine mandates.

**Fig. 2:**
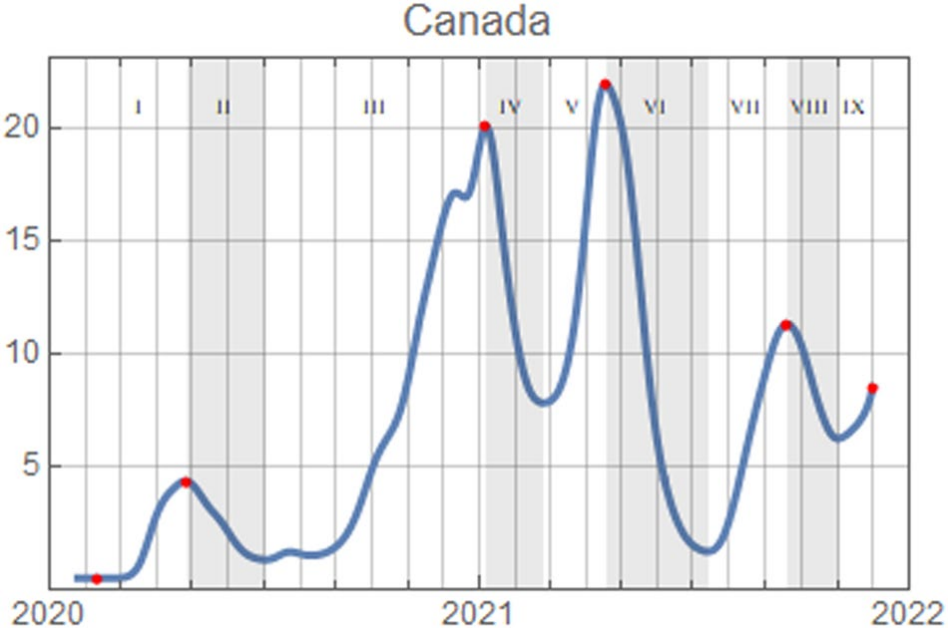
Confirmed cases of Covid-19 per 100 000 population^3^. The strength mitigation implemented in each epoch for each region is shown in Fig. 3. The eight epochs are defined by each successive peak and valley after the start of the pandemic and labeled with the numerals *I*-*VIII*. Epoch *VIII* is defined from the last distinct peak, September 2021, until the end of data.

**Fig. 3:**
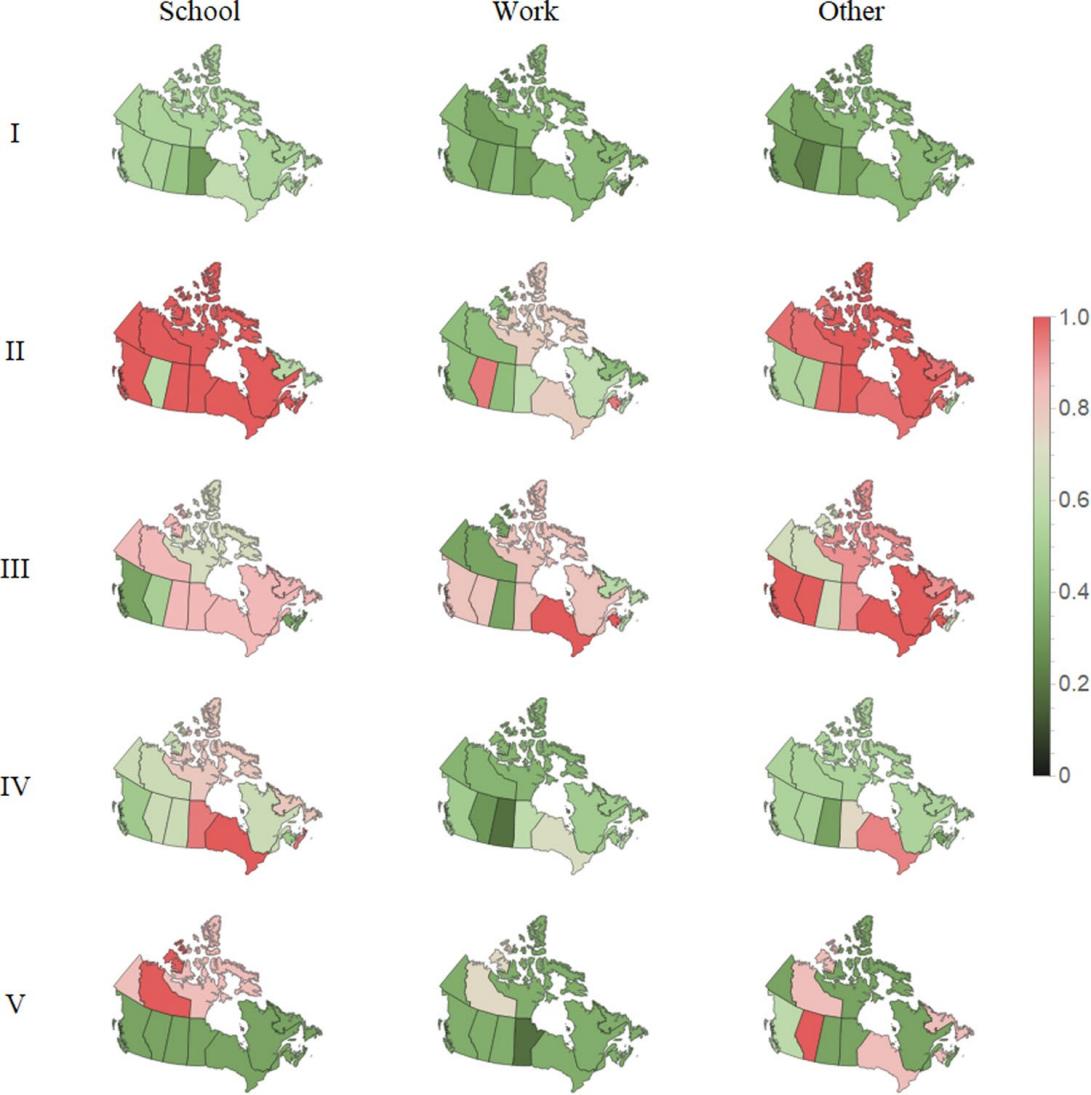
Maps of the Canadian provinces’ and territories’ mean level of mitigation in each of the five epochs between successive peaks in infection, defined in Fig. 2, and three domains of mitigation, School, Work, and Other. The color and intensities of the displayed mitigation are normalized for each domain (school, work, other) over all five epochs (*I*-*V*).

In our repository, we also include NPI timeline figures for each Canadian province and territory. These figures include selected public health measures and provide a visual snapshot of NPIs from March 2020 to January 2022 in Canada. Figure 1 shows the timeline figure for Ontario. The data-set is valuable for visualizing the public health restrictions over the pandemic, with an example provided in Fig. 3, where the eight epochs are defined by each successive peak and valley after the start of the pandemic as seen in Fig. 2. The data-set of public health restrictions is also valuable for mathematical modeling and statistical modeling^5,6,8^.

## Methods

In this section, we describe the data collection process and structure of our database.

### Data collection process

We have started our data collection process by manually gathering all NPIs we could find for every province and territory in Canada from the start of the pandemic using open-access resources, such as government websites and reputable news websites. For every province and territory, a separate excel worksheet has been developed to record all implemented NPIs. These are recorded chronologically from the start of the Covid-19 pandemic. In total, 13 worksheets have been created, and are continuously maintained, for each of the 10 provinces and 3 territories. These worksheets are saved under a single excel file in our Github repository.Refer to Data Sources for the exact document’s location. Every worksheet consists of 2 columns. The first column is “date” and reflects the date of implemented NPI. The second column is “restrictions implemented” and lists the NPIs implemented on that date as well as the corresponding references, outlining where the information was retrieved.

### NPIs selection for data dictionary

Using the collected data on the implemented NPIs we identify three main categories that these measures can be referenced to: school, work and other. The implementations are recorded on a four-level ordinal scale (0–3) for each category. The selection of three domains was intentional so that the data can be easily fit into mathematical models^5,6,8^. The first category includes all restrictions that were enforced in schools. Scheduled school closures are also recorded as level 3 to reflect the level of contact between people in schools even if the reduction in contact was not the result of a public health restriction. The second category includes all the measures that involve workplaces and businesses. The final group was named “other” and includes all the other measures that did not fall into either the school or work category. The following three domains are as follows:

Table 1 presents a decision guide for assigning ordinal values to public health restrictions in various domains. It outlines the ordinal scale and corresponding restrictions for schools, workplaces, and other areas, serving as the reference used to assign an ordinal value representing the level of implemented restrictions. When restrictions were not uniformly implemented across provinces, we prioritize population centers by giving them greater weight in assigning an ordinal value.

**Table 1:** Decision Guide for Assigning Ordinal Values to Public Health Restrictions Dataset.

Domain	Ordinal Scale	Restrictions Guide
School	0	No restrictions
	1	All schools and childcare centres open with some restrictions in place (e.g., physical distancing, use of face coverings, increased cleaning)
	2	Some schools and childcare centres closed, or blended learning implemented (combination of online and in-person classes)
	3	All schools closed for in-person instruction (due to public health restrictions or scheduled holidays)
Work	0	No restrictions
	1	Working from home recommended or most businesses open with some restrictions in place (e.g., physical distancing, use of face coverings, increased cleaning)
	2	Working from home strongly suggested or most businesses closed except for specific sectors or categories of workers
	3	All non-essential workplaces closed or operating remotely, only essential services or businesses remain open (e.g., pharmacies, grocery stores, hospitals)
Other	0	No restrictions
	1	Some minor restrictions on gatherings (e.g., limitations on large gatherings with over 1000 people or gatherings between 101–1000 people), travel not recommended, closure or reduced capacity of some indoor activities, restricted or prohibited visitations to long-term care homes/hospitals. Social distancing, physical distancing, and use of face coverings in public places are either recommended or implemented.
	2	Stricter public gathering restrictions (e.g., limitations on gatherings between 11–100 people), some travel restrictions between provinces, closure or significantly reduced capacity of most indoor activities, closure of some outdoor activities.
	3	Stringent gathering restrictions (e.g., limitations on gatherings of 10 people or less), border closures between provinces for non-essential travel, closure of all indoor activities, and closure of most outdoor activities.

The first category includes all restrictions that were enforced in schools, including scheduled school closures (recorded as level 3) to reflect the level of contact between individuals within schools. The second category encompasses measures pertaining to workplaces and businesses. The final group, referred to as “other,” encompasses all additional measures that do not fall within the school or work category. This includes situations where an area has a significantly higher population of people not at work compared to those who are at work, as well as measures related to home-based activities. Therefore, the “other” category includes measures pertaining to home and any other relevant measures not covered under the school or work categories.

### Recording NPIs

In a separate CSV file, using our timelines and data dictionary, we have constructed time series data for every province and territory in Canada where we capture the differences in the degree of responses from the beginning of the Covid-19 pandemic, starting January 2020.

## Data Records

The database and files described in this manuscript is available from the Harvard Dataverse^4^. The main time series data, which reports the severity of NPIs implemented since the start of the pandemic is presented in CSV format “Provincial_Data.csv”. The data includes a row for each day from the 1st of January 2020 to the 30th of May 2022, with 7 columns: ProvinceName, ProvinceCode, Date, C1 SchoolsClosing, C3 Other, C2 WorkplaceClosing, Vaccine Proof/Passport. The file is continuously updated to reflect the severity of newly implemented NPIs. The data dictionary to this data set has been provided and is available under Data Dictionary.md. Detailed timelines of NPIs for every Canadian province and territory, with references, are located under “Mitigation implementation timeline.xlsx”. This xlsx document includes 13 worksheets (each worksheet represents a province/territory). Each worksheet consists of two columns: date and restrictions implemented. References, outlining where the information was retrieved, are also included in this document and will continue to be updated as pandemic restrictions are implemented or lifted. A GitHub repository that will be updated as the pandemic continues is published at (https://github.com/ddick8/Covid-19-Policy-Response-Canadian-tracker/github.com/ddick8/Covid-19-Policy-Response-Canadian-tracker).

We note that the Oxford Covid-19 Government Response Tracker (OxCGRT)^9^ also contains some NPIs data for Canada. “Datacomparison.xlsx” contains the comparison between our dataset with OxCGRT on two policy indicators: school and work closures. Short notes are provided in the worksheet when differences are found between the two datasets, explaining the reason for our choice of the assigned ordinal.

Timeline figures are stored in “Figures” folder and contain 13 files in JPG format. Each timeline covers the dates from March 2020 to January 2022 and includes selected NPIs.

## Technical Validation

To ensure the validity of the data, we have recorded the sources for each change in the level of public health restrictions in the dataset contemporaneously during the data collection process. Each recorded change in public health restrictions is accompanied by a referenced source in the Excel file ‘Mitigation implementations timelines’ available in the repository and from the Harvard Dataverse^4^. We extensively utilized government resources and credible news agencies to gather information regarding Non-Pharmaceutical Interventions (NPIs). This approach ensures transparency and reproducibility by providing a comprehensive account of the sources and references for each change in the NPI index. It is important to note that while some recorded and sourced changes in public health measures were documented, they did not necessitate a corresponding change in the ordinal scale within the dataset. This documentation enables future researchers to examine the information upon which each ordinal value was assigned, facilitating the questioning of judgments made and the proposal of alternative judgments for each restriction. This documentation enhances the ability to critically evaluate and further analyze the dataset in future research endeavors.

Several international teams have built comprehensive databases that include national and subnational data on public health policies, such as CoronaNet Research Project (CoronaNet)^10^ and Oxford Covid-19 Government Response Tracker (OxCGRT)^9^. To further validate our data, we have run a comprehensive comparison between our dataset and OxCGRT on 2 policy indicators: school and work closures. This comparison is available at the Harvard Dataverse and through our Github repository^4^. There are many similarities but also some notable differences. Both datasets use an ordinal scale for the time series data, mainly 0 to 3 scale. The descriptions of policy indices are similar, but there are a few differences. For example, our tracker accounts for hybrid learning for the school category, while OxCGRT does not explicitly account for this. Therefore, the coding does not match in all instances. To illustrate this, in the province of Ontario on September 8, 2020, it was announced that schools largely resumed in-person learning, but secondary schools were still operating remotely. Therefore, we have chosen to assign 2 to this measure, whereas OxCGRT assigned 1, which is less restrictive. The other difference that was noted is that the timing of interventions was not always the same between the two datasets. This may be due to the use of different resources between the datasets. For instance, according to the provincial government website of Manitoba^11^, as of January 23, 2021, in most of the regions of the province, hair salons, and most retail stores were reopened. Therefore, we have assigned 2 to this measure (middle severity) on this date, whereas OxCGRT had 3 assigned (most restrictive). OxCGRT has amended 3 to 2 a month later, on February 12, 2021. While much of OxCGRT data overlaps with CovidPolicy-Canada, the 2 databases are not the same. The main advantage of using CovidPolicy-Canada is that taxonomy (indices descriptions) is tailored exclusively to Canada. We have also included detailed descriptions of interventions in a separate accessible document so that coding can be easily validated.

## Usage Notes

As public health restrictions are lifted and masking mandates replaced with mask recommendations the careful interpretation of the level of restriction for use in modelling is highlighted. Following the ranking criteria requires that a non-zero restriction level is recorded while masks are still recommended. The recorded restriction level is not necessarily representative of the level of mask use in the population. This data set records the public health mandates and recommendation and does not track the populations compliance with these recommendations.

## Data Availability

The code that is used to generate the Canada and Atlantic bubble weighted average index of public health mitigation is available through the GitHub repository.
